# Brain lesions in oncology patients: recognising metastatic versus non metastatic lesions

**DOI:** 10.1186/1470-7330-15-S1-O40

**Published:** 2015-10-02

**Authors:** Philip Rich

**Affiliations:** 1 Department of Neuroradiology, St George's University Hospitals NHS FT, Consultant Neuroradiologist, The Royal Marsden NHS FT, UK

## 

Therapeutic developments across a range of primary tumours have resulted in more cancer patients with cerebral metastatic disease undergoing active management and imaging surveillance. There has been an increase in the caseload and range of disease presenting to oncology and neuro-oncology MDTs. In this lecture I will present examples of intracranial metastases, discuss differences in appearance according to primary site and tips for differentiating from primary intracranial tumours and non-neoplastic mimics.

